# Cognitive Advantage in Children Enrolled in a Second-Language Immersion Elementary School Program for One Year

**DOI:** 10.5334/pb.469

**Published:** 2019-10-21

**Authors:** Cristina Barbu, Audrey Gonzalez, Sophie Gillet, Martine Poncelet

**Affiliations:** 1 Psychology and Neuroscience of Cognition Research Unit, University of Liège, Liège, BE

**Keywords:** Bilingualism, early immersion education, executive functioning, attentional functioning, improved selective auditory skills

## Abstract

Early bilingualism has been shown to improve attentional and executive functioning. Nicolay and Poncelet (2013a, 2015) have shown that an early immersion program in school of 3 years improves the completion of tasks assessing these skills. This study aimed to determine whether similar benefits might be present after only 1 year of immersion education. The study also observed whether these potential advantages might also have a positive effect on the academic achievement. Participants included 59 immersed children and 57 monolingual controls. The two groups were compared using the same tasks as those employed by Nicolay and Poncelet (2015). The immersed children showed faster responses in comparison to monolinguals on the selective auditory task. No significant differences were observed on the other attentional, executive, or academic tasks. These outcomes suggest that a period of immersion education as short as 1 year can yield cognitive advantages associated with bilingualism.

## Introduction

A large number of studies have shown that early bilingualism acquired in the home or other community settings enhances attentional and executive functioning (for a review see Bialystok, 2011, 2015). These advantages have been observed particularly on tasks requiring conflict resolution and monitoring skills and in different age-grouped populations, such as toddlers, children, young adults, and even older adults. In children, these skills have been measured using different tasks, such as the Attention Network Test for children (ANT; Fan et al., 2002), the Dimensional Change Cart Sort (DCCS; Zelazo, 2006), and the Simon task (Simon & Wolf, 1963). The advantages observed in these tasks have been attributed to the bilingual’s ability to monitor the use of both languages and to inhibit second language (L2) intrusions from the non-target language. More recent findings, however, exhibit contradictory results that revealed null, mixed, or even negative effects (Duñabeitia et al., 2014; Lehtonen et al., 2018; Paap, 2015; Paap & Greenberg, 2013; Paap, Johnson, & Sawi, 2014, 2015; Paap & Sawi, 2016; Valian, 2015). These studies suggest that there are no convincing arguments that would favor the existence of a positive effect of bilingualism on executive functioning and claim that the observed advantages could actually be attributed to other factors, including socio-economic status, video games, music practice, L2 proficiency or L2 onset age (AoA), or language-switching frequency. These factors would account for the inconsistent results observed in the current literature (Boot, Kramer, Simons, Fabiani, & Gratton, 2008; Dong, 2015; Hackman et al., 2015).

Besides studies having investigated cognitive advantages of early bilingualism acquired in the home or other community settings, further studies were interested in examining potential cognitive advantages in children learning an L2 at school using teaching programs, such as the “Content and language – Integrated learning method” (CLIL) (Bialystok & Barac, 2012; Carlson & Meltzoff, 2008; Kalashnikova & Mattock, 2014; Kaushanskaya, Gross, & Buac, 2014; Nicolay & Poncelet, 2013a, 2015; Poarch & van Hell, 2012; Simonis, Van der Linden, Galand, Hiligsmann, & Szmalec, 2019; Woumans, Surmont, & Struys, 2016). One of the key characteristics of the CLIL program is that L2 is not taught as a foreign language. Instead, it is used to teach other academic subjects. Moreover, L2 English classes are taught either by L2 native-like teachers or by individuals with equivalent native-like L2 mastery (Comblain & Rondal, 2001). Since its development in the beginning of 1960s in Canada, the number of schools (in different countries around the world) implementing a CLIL program has increased dramatically (Bjorklund, 1997; O Duibhir, 2009). The CLIL method is of particular interest in bilingual research because it offers the opportunity to assess cognitive L2 effects under particularly homogenous learning conditions. In this program, children enter without any L2 knowledge, receive the same type and quantity of L2 input, and are exposed to similar L2 learning conditions. However, the studies carried out with immersed children, as those conducted on early bilinguals, have yielded divergent results. Some have exhibited a positive effect of the L2 acquired through an early immersive education, while others did not.

First, Nicolay and Poncelet (2013a) found that an L2 acquired through an early immersion education program enhances the development of executive functioning. In this study, the authors compared the performance of two groups of 8-year old children (French-speaking monolinguals) and children enrolled in a 3-year English L2 immersion school program (who were exposed to an L2 only in a school setting) on a series of tasks assessing alerting, divided attention, selective auditory attention, response inhibition, and cognitive flexibility. Immersed and monolingual children were matched in terms of verbal and non-verbal intelligence skills and socio-economic status (SES). The results revealed that immersed children were faster in comparison to monolinguals for all attentional and executive tasks tested, except for the response inhibition task. The authors argued that these attentional and executive processes are highly used and trained when learning an L2. Therefore, they developed during the initial stages of L2 learning. More specifically, alerting skills are needed because children are in a continuous readiness state to process an L2. Selective attention skills are also needed in order to understand and treat L2 linguistic input in class when speaking a language in which the student is not yet automatized or fluent. Furthermore, divided attention skills are also required in order to simultaneously treat L2 auditory and visual information presented in class. Finally, flexibility skills are also used in situations in which children are required to switch from one language to another. Concerning the lack of between-group differences for response inhibition, the authors argued that, compared to balanced bilinguals (who need to exercise continuous control in order to prevent interference from the undesired language over their two constantly activated languages), immersed children are still unbalanced bilinguals and therefore less exposed to L2 production situations in which inhibitory skills might be trained. Moreover, a complementary explanation for the observed results concerns specific aspects related to classroom contexts in which L2 is acquired. In comparison to early bilingual children exposed to both languages at home where constant oral interactions are possible, immersed children might not be frequently exposed to classroom situations in which L2 speaking is needed. This is because, in these settings, there is usually only one teacher for at least 20 pupils, and speaking interactions tend to be fewer compared to persistent one-on-one interactions at home. In a follow-up longitudinal study, Nicolay & Poncelet (2015) controlled for potential differences in attentional and executive functioning prior to enrollment in the immersion program. In this study, French-speaking immersed and monolingual children were tested at the beginning of third kindergarten and at the end of third grade by assessing attentional and executive tasks (except for the response inhibition task) employed by Nicolay and Poncelet (2013a). The results revealed similar findings as the ones observed by Poncelet and Nicolay (2013a), confirming that L2 acquired through an early English immersion program for 3 years enhanced alerting, selective auditory, divided attention, and cognitive flexibility skills. An important aspect to consider for is that Nicolay and Poncelet (2015) replicated their initial findings by using the same experimental design as Nicolay and Poncelet (2013a). Other studies (such as those by Bialystok & Barac, 2012; Carlson & Meltzoff, 2008; Kalashnikova & Mattock, 2014; Kaushanskaya, Gross, & Buac, 2014; Poarch & van Hell, 2012; Woumans, Surmont, & Struys, 2016) have, however, used different tasks in order to assess attentional and executive functioning. This diversity might be a factor explaining the observed non-significant results, which was suggested by related studies (Paap & Greenberg, 2013; Paap, Johnson, & Sawi, 2014; but see Simonis, 2019, non-published doctoral thesis, chapter 5). The aim of the present study was to determine whether the positive attentional and executive effects (alerting, selective auditory attention, divided attention, and cognitive flexibility) observed following 3 years of early immersion education in English might also be observed after only 1 year of immersion education. In order to control for potential confounding within-task factors, the present study employed only attentional and executive measures for which advantages have been shown (Nicolay & Poncelet, 2013a, 2015).

Bialystok and Barac (2012) also found that an L2 acquired through early immersion education has a positive impact on the development of executive (conflict resolution) skills. More specifically, the authors conducted two studies in which they assessed two cohorts of children (grades 2 and 3 for study 1 and grades 2 and 5 for study 2) during the process of learning an L2 in early immersion programs. The subjects had different language backgrounds and language proficiency levels (acquired through the immersion program), and they were immersed in either Hebrew (Study 1) or French (Study 2). They also differed in terms of the length of time spent in the immersion program. Both groups were administered a series of tasks assessing metalinguistic awareness and conflict resolution skills. The results revealed that performance on the conflict resolution task (flanker effect: incongruent RT – congruent RT) positively correlated with the length of time spent within the immersion program. These results seem to suggest that enhanced conflict resolution skills are directly related to the duration of time spent in an immersion program.

In line with these results, Kalashnikova and Mattock (2014) have also shown that early immersion education improves conflict resolution skills. In this study, the performance of 3- to 6-year-old English-speaking monolinguals and English-speaking kindergarteners attending an L2 immersion program in Welsh was compared on the DCCS task. For the immersed group, Welsh used during 75% of the class. English was spoken the rest of the time. The onset age of L2 acquisition differed within the immersed group. More specifically, the younger children (maximum 6 years of age) were exposed to Welsh over 8 months, and for the older children, this exposure lasted for over 20 months. An important factor that should be considered concerns the languages used by the immersed group within the linguistic community. More precisely, the immersed children were living in a bilingual community in which both languages (English and Welsh) were constantly used. This L2 learning environment is very different from the one of a CLIL program in which children are exposed to an L2 only at school. In this context, the immersed group was exposed to the L2 not only within the immersion program, but also beginning from birth or shortly after. This group therefore more frequently encountered situations requiring L2 processing. This supplementary exposure to an L2 may imply an increased recruitment and training of inhibitory skills (inhibition of L2 lexical intrusions). Therefore, this might have contributed to the advantages observed among the immersed children for the conflict resolution task. This advantage was attributed to the constant need and training of immersed children to control the use of the two linguistic systems. In order to speak the target language, one of the languages has to be inhibited for the other to be successfully employed.

In contrast to these studies, Woumans et al., (2016) did not identify a positive effect of early immersion education on conflict resolution skills. Nevertheless, these authors did show that an L2 acquired through early immersion programs enhances non-verbal intelligence skills. During this longitudinal study, two groups of 5-year-old French-speaking children (i.e., subjects immersed in Dutch) and monolingual control peers were matched at the end of 3^rd^ kindergarten (prior to entering in the immersion program) for tasks assessing conflict resolution (or interference inhibition), non-verbal intelligence, SES, and verbal fluency skills. Subjects were tested again using the same tasks after 1 year of immersion education at the end of the first grade. The results revealed a significant positive group effect (more correct responses) with immersed children outperforming monolinguals on a non-verbal intelligence task, but not on one assessing conflict resolution skills. The authors suggested that a short exposure to an L2 in the context of an early immersion program might not be sufficient for the emergence of cognitive advantages relating to executive skills. The positive effect observed on the non-verbal intelligence task was attributed to an improved ability of immersed children to perform analytical reasoning and abstract thinking.

These results partially replicate those of other authors (Carlson & Meltzoff, 2008; Kaushanskaya, Gross, & Buac, 2014; Poarch & van Hell, 2012), who observed that different age ranges of children (from 5- to 7-year-olds) with different language backgrounds immersed in early immersion programs for 6 months to 2 years only within school contexts do not exhibit a cognitive advantage on tasks assessing executive skills (response inhibition, conflict resolution, updating, working memory, and task shifting) when compared to monolingual counterparts. The general explanation provided by such authors (Carlson & Meltzoff, 2008) is similar: a short exposure to an L2 in the context of an early immersion program might not be sufficient to detect cognitive gains related to tasks assessing executive functioning. In this vein, Simonis, Van der Linden, Galand, Hiligsmann, & Szmalec (2019) also failed to find a positive effect of L2 early immersion education on attentional and executive functioning. During this study the authors compared a total sample of more than 500 French-speaking students from Belgium, such as 10 year-old children and 16-year adolescents immersed in English or Dutch for over four years and two groups of monolingual counterparts on different executive control tasks assessing inhibitory control (or interference inhibition), monitoring, switching (or cognitive flexibility skills), and attentional skills. These groups were matched on different control variables including non-verbal intelligence skills and SES levels. Results revealed no significant group differences in neither attentional nor executive task used. Outcomes were attributed to different factors, including insufficient language switching or involvement of immersed children in classroom situations requiring L2 production situations and therefore training of executive control skills. These results however do not align with those of studies suggesting that the degree and length of exposure to an L2 in the context of the immersion program might influence the development of different attentional and/or executive skills (Bialystok & Barac, 2012; Kalashnikova & Mattock, 2014; Nicolay & Poncelet, 2013a, 2015; Poarch & van Hell, 2012). It might be that attentional and executive benefits produced by an L2 immersion education would be observed only during the first phases of the L2 immersion program in which the new L2 requires important attentional resources in order to be treated. In later stages of the L2 acquisition process, L2 might become too automatic as to involve further recruitment and therefore training of attentional and executive skills.

As previously suggested by Nicolay and Poncelet (2013a, 2015), we further predicted that attentional and executive functioning (i.e., alerting, selective, divided attention, and cognitive flexibility) might also be required and, therefore, trained during the first year of the immersion program. Alerting skills might be needed in order to process new or newly acquired L2 information, such as when trying to monitor and understand novel or closely novel L2 information in class. Selective auditory attention might also be required in order to understand new L2 auditory information given by teachers. Divided attention skills might also be involved in situations in which children have to simultaneously pay attention to auditory and visual information (such as when listening to the explanations of the teacher when a new concept is explained orally and visually on the blackboard). Moreover, cognitive flexibility skills might also be solicited in situations in which children are required to switch actively or passively from one language to another between courses taught in only one of the two languages. Consider, however, that for certain schools, weekly courses were given only in one of the two languages (L1 or L2: French or English) with the other language being used the following week. Children enrolled in these schools were therefore not intensively exposed to frequent language switching for training and therefore improving their cognitive flexibility skills.

Besides being associated with bilingualism, improved executive functioning has been shown to predict academic success (e.g., Diamond & Daphne, 2016). More specifically, working memory, inhibition, and shifting or cognitive flexibility have been shown to independently predict math and reading skills from early school years through university years (Best, Miller, & Naglieri, 2011; Cragg & Gilmore, 2014; Cragg et al., 2017; Friso-van den Bos et al., 2013). Cognitive flexibility skills, for instance, are related to arithmetic calculation skills (as assessed by multiple written Arabic digit operations or tasks involving problem-solving). These skills are needed in order to successfully switch among operations, applications strategies, quantity ranges, different types of notations (verbal digits, written Arabic symbols, and nonsymbolic quantity representations), and different stages of a multi-step problem (Bull & Lee, 2014). However, similar findings have not been replicated by other studies. Monette, Bigras and Guay (2011) for instance, observed no correlations between cognitive flexibility skills and mathematics outcomes. These findings indicate that the relationship between improved cognitive flexibility skills and mathematical achievement is not as robust as initially thought, and this needs further investigation.

Recent findings have showed that L2 acquired via an L2 immersion education has a positive effect on academic achievement (as measured by mathematical skills) (Fleckenstein et al., 2019). In this study, German-speaking children following an English CLIL program for 4 years, exhibit better mathematical performance (EMAT; Deutscher Mathematiktest; Gölitz, Roick, & Hasselhorn, 2006) after 1, 2, 3, and 4 years of immersion education as compared to monolinguals German-speaking counterparts comparable in SES and non-verbal reasoning skills. This advantage might be potentially attributed to a more enhanced attentional and executive functioning (alerting, selective auditory, divided attention and cognitive flexibility skills) improved via an L2 immersion education. Better attentional and executive functioning might interfere with the completion of the mathematical tasks proposed and might positively influence performance. During the present study, we tested this hypothesis by trying to establish whether attentional and executive advantages (alerting, selective auditory attention, divided attention, and cognitive flexibility) produced by an L2 immersion education might also positively affect academic performance (as measured by arithmetic assessment). Despite studies showing that different executive skills (i.e., updating, inhibition, and cognitive flexibility) are related to improved mathematical achievement, no study to date has determined whether or not potential cognitive advantages engendered by an L2 acquired through early immersion programs observed in tasks assessing alerting, selective auditory, divided attention and cognitive flexibility skills might also have a positive indirect effect on arithmetic calculation skills. This will be a subsequent aim of the present study. We hypothesized that L2 acquired through a formal one-year CLIL program might have a positive indirect effect on academic achievement based on findings showing that better executive functioning is directly related to bilingualism, but also seem to predict academic success. (Diamond & Daphne, 2016). By extension, positive effects of bilingualism on attentional and executive functioning might also indirectly improve academic achievement. Arithmetic abilities were measured in the present study using one-digit Arabic addition and subtraction operations (de Vos, 1992). We opted for these operations in order to avoid complex linguistic task demands, which might interfere with task performance.

## Methods

### Participants

A total of 116 8-year-old French-speaking children enrolled in first grade participated in the study. Participants were drawn from two language groups: 59 children (28 girls and 31 boys with a mean age of 79 months; range: 73–87 months) enrolled in an English immersion program since the age of 5 (the immersion group) and 57 monolinguals (30 girls and 27 boys with a mean age of 80 months; range: 73–87 months) following a traditional French-learning program (the monolingual group). Monolingual and immersed children spoke French as their native language. Certain participants (15 and 12 from the immersed and monolingual groups, respectively) had members of their families (grand-parents and/or parents and/or siblings) who used an L2 (or several other languages) outside of their home settings. However, these subjects had French as their mother tongue, spoke and were exposed to French only at home, and were not enrolled in extra-curriculum activities (including L2 courses) given an L2. Participants were recruited from different regions of the French-speaking community of Belgium. Immersion participants came from seven schools and monolinguals from 13 schools. In the immersion group, 30 children had 50% of their academic subjects taught in English, and French was spoken the rest of the time. The other 29 children from this group had 75% of academic subjects taught in English, while French was used for the rest of classes. The immersed group learned mathematical and literacy subjects in English (29), French (13), or both languages (17). A part of subjects tested during the present study (30 from a total of 59) were less exposed to their L2 (with 50% of school courses given in L2 during one year) as compared to subjects tested by Nicolay and Poncelet (2013a, 2015), who were all (N = 53 for their 2013 study; N = 51 for their 2015 study) exposed to 75% of school courses in L2 (English) over three years. Note also that the current Belgian system is designed in such a way that only a few schools propose L2 immersion programs (within the French-speaking community of Belgium) with a large exposition to L2 (75% school curricula given in L2). Given this aspect, it is challenging to recruit only subjects exposed to this increased rate of L2. Note also that according to information provided by certain schools weekly courses were given only in one of the two languages (L1 or L2: French or English) with the other language being used the following week.

Non-verbal intelligence skills are likely to influence general learning skills and vocabulary knowledge. French vocabulary knowledge is also likely to affect the development of conceptual abilities, which can influence L2 vocabulary acquisition. Given these assumptions, we have controlled for these factors (non-verbal intelligence skills and French vocabulary knowledge). Moreover, based on previous findings showing that video games, music, and sports practices influence the development of executive functioning (Castel, Pratt, & Drummond, 2005; Best, 2010; Zuk et al., 2014), these different factors have likewise been accounted for. We also determined whether monolinguals and immersed French-speaking children had similar socio-economic status (SES), since SES has been shown to influence the development of executive functioning (da Rosa Picolo et al., 2016).

Immersed children and monolinguals were matched in terms of age, SES, gender, verbal and non-verbal intelligence, video games, music, and sports practice. Detailed outcomes are presented in the “Results” section. Written consent was obtained from parents so that children could participate in the study.

This study was approved by the Research Ethics Committee of the Faculty of Psychology in Liège. We have informed parents of the aim of the study concerning testing conditions (number of testing sessions, location of the testing), assessed cognitive skills, and types of tests used for measurement or assessment. In addition, we provided them with information concerning data confidentiality and subjects’ rights to abandon the study or to be informed of the results.

### Materials and procedures

#### Preliminary measures

*Background questionnaire* – A questionnaire was also given to parents in order to gather information concerning the environment in which the children were living at the time of testing, in addition to their prior history concerning school doubling, SES levels (as indexed by diploma levels obtained of parents), sports, music and video game practice (h/week), general medical history (birth details, psychological, motor, attentional, visual, auditory, or language deficits), and information concerning general language knowledge (L1 and L2 languages used at home and in outdoor settings, as well as motivational reasons for learning an L2). Information gathered using the questionnaire revealed that the subjects had no prior history of school doubling, and they had no psychological, motor, attentional, visual, auditory, or language deficits at the time of testing. Sports, music, and video game practice was measured by asking parents to indicate the frequency with which children were engaged in weekly activities requiring sports, music, and video game practice during the past year. A 5-point Likert scale was used (0 = no practice; 1 = very little or little practice; 2 = mean practice; 3 = frequent practice; 4 = very frequent practice). Parents’ educational levels were indexed as a measure of the SES levels of the children. The two groups (French-speaking immersed and monolinguals) were divided according to the parents’ educational level as reported in the questionnaire into “low” (no professional qualifications at all), “medium” (elementary school qualifications of up to 12 years of years of study), “high” (high qualifications of up to 15 years of study), and “very high” (superior qualifications of at least 17 years of study).

*Non-verbal intelligence* – Non-verbal intelligence was assessed using Raven’s Colored Progressive Matrices (Raven, Court, & Raven, 1998). Children were asked to identify (by pointing to a corresponding image) which one of six pattern segments would correspond at best to a missing segment of a visual-spatial pattern. Raw scores were measured and used for the analysis.

*Receptive vocabulary knowledge* – French-receptive vocabulary knowledge was assessed by using L’Échelle de Vocabulaire en Images Peabody (Dunn, Thériault-Whalen, & Dunn, 1993). Children were required to select (by pointing to the image) which of four line drawings corresponded to a word spoken by the experimenter. The total number of correct responses was measured and introduced into the analysis.

#### English lexical development

*English productive and receptive vocabulary knowledge* – English productive and receptive vocabulary knowledge acquired by the immersed children was measured using two tasks designed to assess these skills. Each measure included a total of 135 items. In the English productive task, children were presented with a picture and asked to name pictures that were presented one by one in English. Items belonged to different categories, such as the human body, geometrical forms, weather forecasts, furniture, clothes, and so forth. If children were not familiar with the items, they were asked to name the picture in French. This procedure was used in order to ensure that they recognized the items. Minor misarticulations were counted as correct provided that the items were phonologically close to the target items. The 135 items used in the productive task were also employed in the receptive task. In this task, subjects were presented with 27 computer images containing five target items each and two distractors (one neutral and one phonologically close). Children were asked to identify the image corresponding to a word spoken in English. Items were presented through headphones. The oral production task was administered first, followed by the receptive version. Synonyms given for target items were considered correct. The total number of correct responses was calculated separately for each task and introduced into the analysis.

### Experimental measures

Alerting, selective attention, divided attention, and cognitive flexibility skills were measured with the Test for Attentional Performance in Children (KITAP – Zimmermann, Gondan, & Fimm, 2002), a computerized standardized battery destined to measure different aspects of attention.

*Alerting* – Alerting was assessed using the sub-test of the Kitap, “The Witch.” In this task, a witch appeared in the middle of the computer screen. Children were asked to press a response key as fast as possible when the stimulus (the witch) appeared. Reaction times were measured and analyzed.

*Selective auditory attention* – Selective auditory attention was measured using the sub-test of the KITAP, “The Owls.” The Owls sub-test is primarily destined to assess divided attention skills. It includes a visual component and an auditory one (see below for the complete description of the divided attention task). An adaptation of this task was employed in order to assess selective auditory skills. Only the auditory component of this task was used. Participants were asked to press a response key as fast as possible only when they detected an irregularity in the sequence (two identical consecutive sounds). Reactions times and errors were assessed and analysed.

*Divided attention* – Divided attention was assessed using the dual task sub-test of the KITAP, “The Owls.” This task was used as a dual measure in order to assess children’s ability to divide attentional resources between two visual and auditory stimuli. In this task, an owl closing its eyes from time to time (visual stimulus) and making squeaky and deep screeches sounds in alternation (auditory stimulus) appeared in the middle of the computer screen. Children were required to press a response key as fast as possible each time they detected an irregularity in the sound sequence (two identical consecutive sounds) and each time the owl closed his eyes. Reaction times and omissions were assessed and analyzed.

*Cognitive flexibility* – Cognitive flexibility was measured using the sub-test of the KITAP “The Dragons house.” Two dragons (one green and one red) appeared simultaneously on the computer screen (each on one side). Participants were required to alternate as fast as possible between the two dragons by pressing a response key corresponding to the side of the screen where the stimulus was located. First, participants were asked to press the key side where the green dragon was, next the side where the red dragon was, and forth so. Acoustic feedback was given when errors were made. Reaction times and errors were assessed and analyzed.

*Arithmetic skills* – Academic achievement was measured by assessing addition and subtraction calculation skills (de Vos, 1992). Children were presented with one sheet (containing one column of addition problems and one column of subtraction problems) and were asked to conduct a maximum number of possible calculations as fast as possible for each given column. Subjects were given 1 minute to solve each column. The total number of correct responses for each type of operation was calculated and analyzed.

### General procedure

Subjects were tested in their respective school between January–May. All tasks were administered in a fixed order in French during two individual sessions, which lasted approximately 1 hour each. As a comparison for the first testing session, the second session was conducted at an interval of 7 to 15 days.

During the first session, different skills were measured: alerting, selective auditory attention, non-verbal intelligence, and English vocabulary knowledge. During the second testing session, the following skills were assessed: divided attention, cognitive flexibility, receptive vocabulary knowledge, and arithmetic skills. Stimuli were presented on a laptop with a standard screen dimension. Children were seated at a comfortable distance from the computer screen.

### Statistical procedure

The performance of participants on the experimental measures was compared using t-tests (independent sample t-tests) and chi-squared tests. We also used Bayesian t-tests in order to control for biases related to the normal distribution of data, the null hypothesis, statistical power, or p values (Wagenmakers, 2007; Wagenmakers et al., 2015). This approach allows for a comparison of two models by reflecting a group effect compared to a null model in which no group effect is present using the Bayesian factor. This factor reflects the probability of occurrence for these two models. The level of significance of the Bayesian factor is not related to a threshold value as in inferential statistics. It is, however, generally acknowledged that a Bayesian factor greater than 3 is considered to be moderate evidence, a Bayesian factor over 10 is strong evidence, and a Bayesian factor higher that 30 is considered to be very strong evidence (Lee & Wagenmakers, 2014).

## Results

### Preliminary measures

Chi-square tests revealed that the two groups were similar in terms of gender, X^2^ (1) = 0.31, p = 0.57; SES, X^2^ (3) = 3.13, p = 0.37. Chi-square tests also showed that immersed and monolinguals were comparable in terms of sports, X^2^ (4) = 3.80, p = 0.43; music, X^2^ (3) = 1.64, p = 0.64; and video game practice, X^2^ (4) = 0.88, p = 0.92. T-tests revealed no significant group differences in terms of age (t(114) = –0.90; p = 0.36), receptive vocabulary knowledge (t(114) = –0.65; p = 0.51), non-verbal intelligence (t(114) = 0.02; p = 0.97. For the Bayesian t-tests, the results revealed that the Bayes factors for the alternative model (including a group effect) were only 0.28 for age, 0.24 for receptive vocabulary knowledge, and 0.19 for non-verbal intelligence. These results offer no significant evidence for a group difference in these different control measures. Descriptive, inferential, and Bayesian statistics, as well as mean comparisons are presented in Tables 1, 2 and 3.

**Table 1:** Descriptive, inferential, and Bayesian statistics, as well as mean comparisons for age, receptive vocabulary knowledge (for a French-receptive vocabulary), and non-verbal intelligence.

	Immersed	Monolinguals	Inferential statistics		Bayesian statistics			

	N = 59 Mean (SD)	N = 57 Mean (SD)	T-Value	p	BF_10_	BF_10_ (error %)	BF01	BF01 (error %)

Age (months)	79.47 (3.67)	80.07 (3.42)	–0.90	0.36	0.28	0.004	3.51	0.004
French-receptive vocabulary (max = 170)	83.10 (12.51)	84.87 (16.39)	–0.65	0.51	0.24	0.001	4.17	0.001
Non-verbal intelligence (max = 36)	22.33 (4.40)	22.31 (4.48)	0.02	0.97	0.19	0.001	5.06	0.001

**Table 2:** Contingency table for video game, sport and music practice.

	Video game practice X^2^ (4) = 0.88, p = 0.92					Sport practice X^2^ (4) = 3.80, p = 0.43					Music practice X^2^ (3) = 1.64, p = 0.65				

	0	1	2	3	4	0	1	2	3	4	0	1	2	3	4
I	12	10	20	14	3	6	3	9	25	16	48	1	4	6	0
N	14	11	15	14	3	8	0	7	28	14	49	0	2	6	0

Legend: I = immersed; M = monolinguals; 0 = no practice; 1 = very little or little practice; 2 = mean practice; 3 = frequent practice; 4 = very frequent practice.

**Table 3:** Contingency table for SES status.

	SES status X^2^ (3) = 3.13, p = 0.37			

	0	1	2	3
I	0	20	23	16
N	1	26	16	14

Legend: I = immersed; M = monolinguals; 0 = low (no professional qualifications at all); 1 = medium (elementary school qualifications of up to 12 years of years of study); 2 = high (high qualifications of up to 15 years of study; 3 = very high (superior qualifications of at least 17 years of study).

L2 lexical skills (English-productive and English-receptive vocabulary knowledge) acquired during the immersion program was also measured after 1 year of immersion education. This procedure was applied only for the immersed group in order to ensure that these subjects were successfully acquiring L2 vocabulary during the first year of the immersion program. The population examined in the present study acquired an L2 vocabulary level similar to the one obtained by children immersed for 1 year, who were tested in the study of Nicolay and Poncelet (2013b).

In terms of English-productive vocabulary knowledge, our subjects were able to produce orally between 11 and 103/135 correct items. As for their English-receptive vocabulary skills, they were capable of recognizing between 42 and 130/135 items correctly. Descriptive statistics are presented in Table 4.

**Table 4:** Descriptive statistics for the immersion group for English vocabulary knowledge.

	Mean (SD)	Range

Productive English vocabulary knowledge (Max = 135)	40.69 (21.90)	11–103
Receptive English vocabulary knowledge (Max = 135)	92.30 (22.50)	42–130

During the English oral vocabulary task, children produced the target stimulus, a synonym, or the French word for the item when the item was unknown in English. Alternatively, they had no response. During the English-receptive vocabulary task, children named the image corresponding to the correct item or selected a random image when the item was unknown. Very few cases of phonological distractions (similar phonological items produced instead of the target items) were observed.

### Experimental measures

#### Attentional and executive measures

A series of t-tests were conducted in order to compare the performance of immersed and monolingual groups in terms of reaction times, errors, and omission rates on measures of alerting, selective auditory attention, divided attention, and cognitive flexibility. T-tests (Love et al., 2015; https://jasp-stats.org/) carried out on accuracy data revealed no significant group differences for the selective auditory attention task (p = 0.87; range for immersed children : 0–20 errors; range for monolinguals: 0–22 errors; mean errors for immersed children : 4.67 ± 4.47; mean errors for monolinguals: 4.80 ± 4.41), for the divided attention task (p = 0.11; range for immersed children: 0–21 omissions; range for monolinguals: 0–20 errors; mean omissions for immersed children : 5.20 ± 4.81; mean omissions for monolinguals: 6.59 ± 4.72), or for the cognitive flexibility task (p = 0.16; range for immersed children: 0–11 errors; range for monolinguals: 0–12 errors; mean errors for immersed children : 3.61 ± 2.83; mean errors for monolinguals: 4.33 ± 2.68). Moreover, we observed a no speed-accuracy trade-off for this later task as provided by a correlation analysis conducted between response speed and error rates (r = 0.08; p = 0.36). The t-tests revealed a significant group difference in terms of response speed for the selective auditory task, t(114) = –2.12 (p = 0.03) with immersed children performing faster in comparison to monolingual peers. However, no significant group differences were observed in terms of response speed on measures of alerting (t(114) = –0.17; p = 0.86), divided attention (t(114) = –0.41; p = 0.67), and cognitive flexibility (t(114) = 1.51; p = 0.13). These results were also confirmed by Bayesian t-tests, which revealed that the alternative model (including a group effect) for the selective auditory task was 2.9 times more likely than the null model (including no group effect). These results suggest that an early L2 immersion education of 1 year enhances selective auditory attention. As for the other attentional and executive tasks, the results revealed that the alternative model was only 0.22 for alerting, 0.27 for divided attention, and 0.08 for cognitive flexibility. Moreover, the null model (supporting no group difference) was 4.42 for alerting, 0.34 for selective auditory attention, 3.59 for divided attention, and 11.85 for cognitive flexibility. These findings offered a significant evidence for a positive effect of an L2 acquired through a 1-year early immersion program on selective auditory attention but not on alerting, divided attention, and cognitive flexibility.

We have also employed additional t-tests in order to establish if there were potential differences according to children’s L2 degree of exposition (29 children had 75% of academic subjects given in L2, and 30 children had 50% of academic subjects given in L2). Results revealed no significant group differences in terms of children’s degree of L2 exposure for either alerting (p = 0.36), selective auditory (p = 0.85), divided attention (p = 0.95), or cognitive flexibility skills (p = 0.84). This analysis was conducted after controlling for age, gender, SES status, gender, video game, sports and music practice, and non-verbal reasoning skills.

Descriptive inferential statistics, Bayesian statistics, and mean comparisons for measures of alerting, selective auditory attention, divided attention, and cognitive flexibility are presented in Table 5.

**Table 5:** Descriptive, inferential, and Bayesian statistics for measures of alerting, selective auditory attention, divided attention, and cognitive flexibility.

	Immersed N = 59 Mean (SD)	Monolinguals N = 57 Mean (SD)	Inferential statistics			Bayesian statistics			

			T-Value	p	Effect Size (Cohen’s d)	BF_10_	BF_10_ (error %)	BF01	BF01 (error %)

Alerting (RT)	375.62 (91.06)	378.17 (67.05)	–0.17	0.86	0.00	0.22	~0.021	4.42	~0.021
Auditory attention (RT)	817.20 (141.93)	875.36 (152.49)	–2.12	0.03*	0.03	2.90	~0.003	0.34	~0.003
Divided attention (RT)	774.89 (90.24)	782.66 (110.82)	–0.41	0.67	0.00	0.27	~3.474e -5	3.59	~3.474e -5
Cognitive flexibility (RT)	1366.62 (331.58)	1277.52 (299.57)	1.51	0.13	0.01	0.08	~0.003	1.85	~0.003

df = 114. RT = Reaction times. BF_10_ = Bayes factor for the alternative hypothesis vs. the null hypothesis. BF_01_ = Bayes factor for the null hypothesis vs. the alternative hypothesis.

#### Arithmetic measures

Inferential t-tests revealed a marginal group difference in favor of monolinguals in terms of addition operations (t(114) = –1.93; p = 0.055). No significant group difference was observed for subtraction operations (t(114) = –0.45; p = 0.64). However, Bayesian t-tests revealed that the alternative model that included a group effect was only 1.05 for additions and 0.21 for subtractions. This analysis also showed that the null model, including the no effect group, was 0.95 for additions and 4.60 for subtractions. This provides anecdotal evidence. Descriptive, inferential, and Bayesian statistics as well as mean comparisons are presented in Table 6.

**Table 6:** Descriptive, inferential, and Bayesian statistics for subtraction and addition operations.

	Immersed N = 59 Mean (SD)	Monolinguals N = 57 Mean (SD)	Inferential statistics			Bayesian statistics			

			T-Value	p	Effect Size (Cohen’s d)	BF_10_	BF_10_ (error %)	BF01	BF01 (error %)

Arithmetic skills-Addition (CR)	7.84 (2.37)	8.80 (2.94)	–1.93	0.055	0.03	1.05	~4.589e -6	0.95	~4.589e -6
Arithmetic skills-Subtraction (CR)	6.50 (2.91)	6.75 (2.86)	–0.45	0.64	0.00	0.21	~2.474e -5	4.60	~2.474e -5

df = 114. CR = correct responses. BF_10_ = Bayes factor for the alternative hypothesis vs. the null hypothesis. BF_01_ = Bayes factor for the null hypothesis vs. the alternative hypothesis.

## Discussion

The main aim of the present study was to determine whether cognitive advantages observed on tasks assessing different attentional and executive functions (alerting, selective auditory attention, divided attention, and cognitive flexibility skills) acquired through a 3-year early immersion education program (Nicolay & Poncelet, 2013a, 2015) would also be observed after only 1 year of immersion education. We hypothesized that similar attentional and executive benefits would be found after only 1 year of immersion education given that these skills are likely to be required and, therefore, potentially trained and improved during the first year of the immersion program. Any non-significant group difference observed would suggest that a short exposure of only 1 year to an L2 would not be enough for cognitive advantages on these tasks to emerge. If this was the case, such results would support previous findings (Carlson & Meltzoff, 2008; Kaushanskaya, Gross & Buac, 2014; Poarch & van Hell, 2012) showing that reduced exposure to L2 through immersion education programs do not produce attentional and executive benefits. Another aspect to consider concerning the above-mentioned studies is that they have used different tasks when assessing attentional and executive functioning. Such divergent task usage might have explained the observed non-significant effects, which is a hypothesis supported by previous related findings (Paap & Greenberg, 2013; Paap, Johnson, & Sawi, 2014). In order to control for potentially confounding within-task factors, the present study employed only attentional and executive measures for which advantages have been previously shown (Nicolay & Poncelet, 2013a, 2015). Furthermore, the second aim of the present study was to determine whether attentional and executive skills (i.e., alerting, selective auditory attention, divided attention, and cognitive flexibility skills) that are potentially improved through an early immersion education of 1 year might also have a positive and indirect influence on academic achievement and, more specifically, on mathematical skills. We hypothesized that L2 acquired through a formal one-year CLIL program might have a positive indirect effect on academic achievement based on findings showing that better executive functioning is directly related to bilingualism, but also seem to predict academic success. (e.g., Diamond & Daphne, 2016). By extension, positive effects of bilingualism on attentional and executive functioning might also indirectly improve academic achievement.

The results of the present study revealed a significant group difference in terms of selective attention tasks, but not on tasks measuring alerting, divided attention, and cognitive flexibility. Moreover, additional t-tests performed according to immersed subject rate of L2 exposition (30 subjects with 50% of academic subjects given in L2 versus 29 subjects with 75% of academic subjects given in L2) revealed no significant group differences for any applied attentional and executive tasks. These results do support other previous findings, which also showed that a reduced exposure (of only one year) to an L2 does not produce executive advantages. One potential explanation for the lack of group differences is that children were not sufficiently exposed during the first year of the immersion program to L2 activities involving these skills so as to develop a cognitive advantage compared to selective attention skills which are likely to be more required during the first year of the immersion program for activities requiring oral comprehension. More precisely, concerning the alerting task, immersed children tested in the present study were probably not sufficiently exposed to L2 processing situations requiring an alerting state so as to develop a cognitive advantage in this regard. Furthermore, a second possible explanation for this result could be that the task used to assess these skills was not cognitively demanding enough for any group difference to be detected. Previous studies have indeed suggested that cognitive bilingual advantages would be observed only by employing complex and demanding cognitive tasks (Costa et al., 2009). An important factor to consider concerning the alerting task employed is that this task is a very simple, perceptual response speed measure because it requires participants to respond by pressing a response key when a simple visual stimulus (a witch) appears in the middle of the computer screen. Basic perceptual processing of visual stimuli via motor responses was also needed for the other attentional and executive tasks because they required participants to press a key as fast as possible in response to visual stimuli appearing in the middle of the computer screen. These results indicate that the advantage observed for the selective auditory attention task cannot be explained by a potential non-observed response speed advantage induced by treating simple visual stimuli presented on screen.

A possible complementary explanation that accounts for the lack of the observed between-group differences on the divided-attention task is that during the year of the immersion program, attentional resources might be limited (Scalf et al., 2013). Therefore, it is possible that children are required to focus their attention mostly on one type of sensory input (auditory). Teachers were likely presenting information to children primarily visually or auditory. A second explanation could be that immersed children were not frequently exposed to situations requiring the simultaneous treatment of visual and auditory stimuli. As for the cognitive flexibility task, a possible explanation for the lack of observed between-group differences could be that immersed French-speaking children were not switching frequently enough (passively or actively) between languages in the classroom setting or outdoor activities for group differences to be detected. According to information provided by teachers and parents, children were rarely switching between languages during the first year of the immersion program. At this moment, weekly courses were taught either in one language with the other language being used the next day. In this context, immersed children were not frequently faced with opportunities requiring an extensive training of their cognitive flexibility skills. Several authors (Bialystok & Barac, 2012; Carlson & Meltzoff, 2008; Kaushanskaya, Gross, & Buac, 2014; Poarch & van Hell, 2012; Puric, Vuksanovic, & Chondrogianni, 2017) have suggested that attentional and executive skills develop only with increasing exposure to L2. Immersed children tested in the present study were still poorly proficient bilinguals and, therefore, they did not have the opportunity to intensively use and train their attentional and executive skills. As suggested by previous authors, longer exposure to L2 would probably be necessary in order to detect advantages on tasks requiring executive skills. These results do support other previous findings, which also showed that a reduced exposure to an L2 does not produce executive advantages (e.g., Carlson & Meltzoff, 2008; Kaushanskaya, Gross, & Buac, 2014). The absence of a significant group’s difference as compared to Nicolay and Poncelet (2013a, 2015) results cannot be explained by children’s degree of L2 exposure given that no significant group difference was observed between children exposed to 50% or to 75% of L2 courses given in L2. In this context only the length and not the rate of exposure (of one year) to L2 can explain the significant positive effect observed in immersed children as compared to monolinguals. Note also that as compared to immersed children tested by Nicolay and Poncelet’s (2013a, 2015) which were all exposed to 75% of school courses in L2, 30 (from a total of 59) of our immersed children had only 50% of school courses in L2. Moreover, Nicolay and Poncelet’s (2013a, 2015) subjects were exposed for a longer period to their L2 (for over 3 years) as compared to immersed subjects tested during the present study enrolled for only one year within the immersion program.

Despite a lack of group differences for tasks assessing alerting, divided attention, and cognitive flexibility, a positive effect was observed in favor of immersed children on the selective auditory attention task. This advantage cannot be explained by age, gender, verbal and non-verbal intelligence, SES, or video game, music, and sports practice because all of these factors have been accounted for. These results support the findings of Nicolay and Poncelet (2013a, 2015) and confirm our initial hypothesis that an L2 acquired through an early immersion education program of 1 year has a positive effect on auditory attention skills, likely because these skills are required and thoroughly trained. These skills are likely to be required and trained during the first year of the immersion program in situations in which new, barely new, or old L2 acoustic information have to be constantly treated (e.g., decoded, encoded) by pupils in class. In comparison to monolinguals taking traditional French classes, who have to focus their auditory attention skills only on the comprehension of academic subjects taught in a language that is highly automatized and fluent (French in the present case), immersed children are faced with the constant challenge of understanding complex academic subjects in a language not yet mastered and automatized (English in the present case). This leads to the continuous use and training of their auditory attention skills. Therefore, auditory attention skills are likely to be much more commonly used in comprehension activities during the first year of the immersion program compared to alerting, divided attention, and cognitive flexibility skills, which are likely not required as much.

Since previous studies have shown that improved executive functioning predicts better academic achievement, the second aim of the present study was to determine whether potential improvements in attentional and executive skills acquired through an early immersion education of 1 year could also have a positive effect on academic performance. Academic performance was assessed by an addition and subtraction operation sub-task (de Vos, 1992). Results revealed a marginal group difference in favor of monolinguals in terms of addition operations with no significant group difference being observed for subtraction operations. However, Bayesian t-tests showed that the alternative model that included a group effect was only 1.05 for additions and 0.21 for subtractions. These results provides anecdotal evidence. These results suggest that even if an early L2 immersion education of only 1 year has positive effects on selective auditory attention, this advantage will not automatically enhance mathematical achievement. In order to further explore the present results larger samples of monolinguals and immersed should be eventually compared.

A possible explanation for these results is that at the end of the first year of the immersion program, children are still in the process of acquiring basic mathematical skills. In this context, a high level of heterogeneity relating to performance skills might still be present. Secondly, a large proportion of the immersion population tested in the present study learned addition and subtraction operations in both languages (English and French; N = 17) or only in the L2 program (English; N = 29). It is possible that subjects learning these operations in two codes were potentially faced with an increased cognitive load engendered by the concomitant activation of the two linguistic codes (English and French) of bilinguals. Moreover, subjects who learned these abilities in both languages were frequently exposed to two linguistic codes (English and French). It might be that these codes were also simultaneously activated while subjects conducted the addition sub-component of the task. This concomitant activation might have reduced the task completion speeds of participants. This hypothesis is supported by the findings of Magiste (1980), who showed that German-Swedish bilinguals learning both languages concomitantly also display increased response latencies and error rates in comparison to monolinguals on a task assessing subtraction and addition operations. This disadvantage was explained in terms of a language interference in bilinguals related to the parallel activation of two language systems when performing arithmetic operations. Performance advantages on tasks assessing arithmetic skills might be potentially observed in later stages of the L2 acquisition process in which L2 is better mastered and automatized. In these stages, participants might have better control over their two languages and, therefore, might be faced with less interference between the two. Moreover, other potentially contributing factors involved in the acquisition of basic arithmetic skills might also explain the performance of subjects on this task. These factors include training methods used to train arithmetical skills, time spent using these abilities (within and in outdoor school contexts), and the motivation of children to learn these skills.

In conclusion, the results of the present study suggest that a short exposure to an L2 acquired through an early immersion education of only 1 year has a positive effect on selective auditory attention skills, at least while these skills are extensively used and trained. However, this advantage might not have a positive and indirect effect on academic achievement (as assessed by addition and subtraction operations).

Future studies should further explore these findings by testing a broader range of arithmetic tasks. Moreover, the results of the present study revealed that an L2 acquired through an immersion program of 1 year does not seem to have a positive influence on alerting, divided attention, and cognitive flexibility skills. As suggested by previous authors, a longer exposure to L2 might be required to observe cognitive advantages on these tasks. Future studies should investigate this issue in order to determine whether the same benefits might also be present in 2nd-grade children enrolled in early immersion programs. This will be the aim of a forthcoming study.
