# Correction: Cognitive Advantage in Children Enrolled in a Second-Language Immersion Elementary School Program for One Year

**DOI:** 10.5334/pb.532

**Published:** 2020-01-27

**Authors:** Cristina Barbu, Audrey Gonzalez, Sophie Gillet, Martine Poncelet

**Affiliations:** 1 Psychology and Neuroscience of Cognition Research Unit, University of Liège, Liège, BE

## Abstract

This article details a correction to the article: Barbu, C., Gonzalez, A., Gillet, S. and Poncelet, M., 2019. Cognitive Advantage in Children Enrolled in a Second-Language Immersion Elementary School Program for One Year. *Psychologica Belgica*, 59(1), pp. 416–435. DOI: http://doi.org/10.5334/pb.469

## Correction

The publication Barbu et al (2019) included an error within the first sentence of the Methods section, providing an incorrect age group for the participants.

The original sentence was:

A total of 116 8-year-old French-speaking children enrolled in first grade participated in the study.

This should instead read:

A total of 116 73 to 87 month-old French-speaking children enrolled in first grade participated in the study.

The authors have brought this error to the editor’s attention.
